# A Multiscale Region-Based Motion Detection and Background Subtraction Algorithm

**DOI:** 10.3390/s100201041

**Published:** 2010-01-28

**Authors:** Parisa Darvish Zadeh Varcheie, Michael Sills-Lavoie, Guillaume-Alexandre Bilodeau

**Affiliations:** Departement of Computer and Software Engineering, École Polytechnique de Montréal, P.O. Box 6079, Station Centre-ville Montréal (Québec), H3C 3A7, Canada; E-Mails: michael.sills-lavoie@polymtl.ca (M.S.L.); guillaume-alexandre.bilodeau@polymtl.ca (G.A.B.)

**Keywords:** motion detector, background subtraction, iterative subdivision, region-based, histograms, Gaussian Mixture

## Abstract

This paper presents a region-based method for background subtraction. It relies on color histograms, texture information, and successive division of candidate rectangular image regions to model the background and detect motion. Our proposed algorithm uses this principle and combines it with Gaussian Mixture background modeling to produce a new method which outperforms the classic Gaussian Mixture background subtraction method. Our method has the advantages of filtering noise during image differentiation and providing a selectable level of detail for the contour of the moving shapes. The algorithm is tested on various video sequences and is shown to outperform state-of-the-art background subtraction methods.

## 1. Introduction

To track moving objects in videos, two main approaches are possible: explicit segmentation of the moving regions following by matching of the segmented regions, or searching of moving objects based on appearance without segmentation. Although segmentation is known to be challenging, segmenting moving regions makes it possible to focus on a search by appearance on a smaller area. Furthermore, any additional information is always welcome in computer vision. This is why background subtraction is an important subject and forms the basis of many algorithms and applications in video surveillance to detect suspicious behaviors of people or objects, and in human-computer interactions (HCI) to recognize the posture, gestures, or activities of people so that the environment can react accordingly.

Even with all the effort made to date, background subtraction, which is applicable to still camera images, continues to face a number of difficulties. The principle behind background subtraction is to subtract and threshold a background model image from the current frame. The result gives the differences between the two subtracted images, and it is hypothesized that these differences correspond to moving objects. In practice, this is not always the case, as differences may correspond to shadows, changes of lighting, or camera noise. Furthermore, some of them may correspond to changes in an image, like waving leaves or waves on a lake, which are irrelevant to the application. The challenge, then, is to propose a background model that allows filtering of these unavoidable perturbations, while still correctly detecting the moving objects of interest.

Many background subtraction methods have been proposed with different models and update strategies. Most rely on the difference between individual pixels. Since perturbations often affect individual pixels, this may cause misdetection when performing differentiation, as observed in [1]. Our hypothesis is that using a neighborhood around a pixel should allow the filtering of perturbations that affect only a few pixels in a region. We propose a method where background subtraction is performed iteratively from large to small rectangular regions using color histograms and a texture measure. In addition, the classical Gaussian Mixture method [2] is used at the smallest region level to improve the results even more. In order to give more accurate distance measures, which account for the error distribution among the histogram bins [3], the Minimum Difference of Pair Assignments (MDPA) distance is applied on the histograms. This algorithm and its analysis constitute the contribution of this paper.

We thoroughly tested our method by comparing detected moving regions with ground-truth regions using true and false positive rate measures. We also characterized the impact of parameter change on the results to evaluate parameter sensitivity and stability. The results show that our proposed method, combined with Gaussian Mixture, outperforms Gaussian Mixture alone and other state-of-the-art methods.

**Figure 1.**
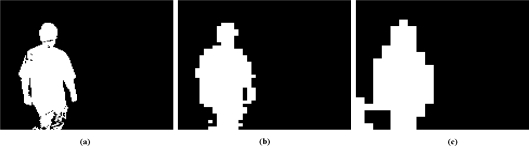
Motion detection at different scales. Finest rectangle size of. (a) 4 × 3; (b) 16 × 12; and (c) 32 × 24.

One of the advantages of our proposed approach compared to state-of-the-art methods is that it reduces the number of false detections, as pixel-level differentiation can be performed in regions with significant motion only. Another advantage is that the subdivision of large regions into small ones can be stopped before pixel level is reached. So, if required, only a coarse background subtraction need be performed (see Figure 1).

The paper is structured as follows. Section 2. gives an overview of the state-of-the-art of background subtraction methods. Section 3. presents the proposed motion detection and background subtraction algorithms, and Section 4. demonstrates the capability of the proposed method with a thorough analysis. Finally, Section 5. concludes the paper.

## 2. Related Work

Most background subtraction methods consider pixels individually. One of those most often used is the Single Gaussian method [4], in which statistics (mean and standard deviation) are computed for each background pixel. A pixel is considered a foreground pixel if its value when compared to its mean is larger than a threshold based on the standard deviation. This model does not deal with multimodal background color distribution, and thus it cannot handle well scenes with swaying vegetation or rippling water, as it assumes a static background. Another method with a similar drawback is based on the mean or the median of individual pixels [5]. This is the temporal average, which simply takes the average RGB values of each background pixel over a certain number of frames and performs a comparison with a threshold (not based on variance). In this work, that method is explored by testing variations on how the background is updated. The authors suggest a different method for selectively updating the pixels, so that only pixels corresponding to background are updated.

The Single Gaussian method can be improved significantly by using more than one Gaussian per pixel [2]. In this case, the *k* best distributions are selected dynamically, and a pixel is to be labeled as foreground if it is different from the *k* distributions based on standard deviation and mean. Both the Single Gaussian and Gaussian Mixture models can handle illumination change by updating dynamically the distributions dynamically. Many authors have proposed improvements to this algorithm; for example, for updating the mixture model [6], or for dynamically adapting the number of distributions to consider for each pixel [7]. Furthermore, the work of Chen *et al.* [8] improves the Gaussian Mixture approach by using a stochastic approximation procedure to estimate the model parameters and to obtain an optimal number of mixture components.

A related model uses the median, the minimum, and maximum values of a pixel [9]. A foreground pixel is labeled based on its difference from the maximum and the minimum values relative to the median of the difference between consecutive pixel values. An advantage of this method is that, as it is median-based, it can learn the background model even if there is motion in the scene as it is median-based.

Edges can be used to model the background instead of the pixel colors, for example, edge histograms for pixel blocks may be used to model the background [10]. In another work [11], both color and edge information are used for background modeling and for subtraction. For color information, a Single Gaussian model is used, whereas for edge information, a sobel edge detector is applied to each color channel and then the mean and variance of the edge strength are computed to devise a second Single Gaussian model.

A Bayesian approach is proposed in the work of Li *et al.* [12]. Spectral features (color information), spatial features (statistics on pixel regions), and temporal features (filters to model temporal change of pixels) are incorporated in a Bayesian framework to characterize the background appearance at each pixel. The background is represented by the most significant and most frequently occuming features.

In the work of Wu *et al.* [13], the authors propose the use of ratio images as the basis for motion detection. The effects of illumination changes are filtered, because static pixels change their values in a similar way, while the presence of a foreground object alters this uniformity in the ratio image.

With the Gaussian Mixture method, it may not be possible to easily model backgrounds with fast variations accurately with a few Gaussians. To overcome this problem, a non-parametric approach, which estimates the probability density function of each pixel using a kernel density estimation technique, was proposed in [14]. The probability of observing pixel intensity values is estimated based on a sample of intensity values for each pixel. This method can adapt very rapidly to change in the background, which makes it very sensitive for detecting moving targets. However, it is not applicable when long durations are needed to sufficiently sample the background (significant wind load on vegetation, for example) because of memory constraints. To overcome this problem, another method, called CodeBook [15], has been proposed. This method represents, in a compressed form, the background model for a long image sequence under memory constraints. Its algorithm adopts a clustering method to construct the background model, and builds a codebook for each pixel with one or more codewords. Based on the color distortion, samples at each pixel are clustered. Detection is performed by comparing the current image with the background model with respect to color and brightness differences.

Some region-based methods have also been proposed. Recently, a method based on local binary patterns was tested [1]. This method, called TBMOD (Texture-based Moving Object Detection), models each pixel using a binary pattern computed by comparing the neighboring pixel values with a center pixel. The binary pattern indicates, for each neighboring pixel, if it is smaller or larger than the center pixel. Such binary patterns are calculated in a circular region around a given pixel, and the binary pattern distribution in the circular region and over time is used to model that pixel. Thus, foreground labeling decisions are based on a region around a pixel and pattern similarity. However, since a center pixel is used as the reference for computing the binary pattern, a perturbation on this particular pixel can lead to misdetection.

The methods presented in the work of Matsuyama *et al.* [16] and Mason and Duric [10] are also related to our proposed method. In the work of Matsuyama *et al.* [16], change detection is achieved by correlation between blocks of fixed size in the image. Foreground is thus detected by correlating of corresponding blocks. Because it uses correlation, this method may have difficulties with some dynamic scene and with local noise in blocks. Furthermore, fixed block size requires a compromise for object size and noise filtering. In the work of Mason and Duric [10], fixed block size and edge histograms are used. Using fixed block size makes it harder to balance robustness to noise and detection precision. Edges do not permit the filtering of noise coming from small motions, like waving leaves. Finally, the method of Bourezak *et al.* [17] uses blocks at different scales and color histograms. Our proposed method is an improvement over this method, in that it integrates texture and the Gaussian Mixture model.

## 3. Methodology

Like all background subtraction approaches, our proposed method is composed of a regularly updated background model and a similarity measure to compare a given frame with that background model. The background is modeled at different scales with color histograms of rectangular regions. We use histograms to model the regions, because they are robust to local noise and because histograms of larger regions can be recursively built from the combination of smaller ones. This is not true for the descriptors that account for pixel locations, however. Thus, histograms can be computed rapidly and can filter noise. The current frame in which we want to detect motion is modeled in the same way. Motion is detected by comparing the corresponding rectangular regions from the coarsest scale to the finest scale. Comparisons are performed at a finer scale only if motion is detected at a coarser scale.

To devise a more efficient method, the Gaussian Mixture method is applied at the finest scale. We call our method RECTGAUSS-Tex.

### 3.1. Rectangular Region Modeling

#### 3.1.1. Background model and updating

**Figure 2.**
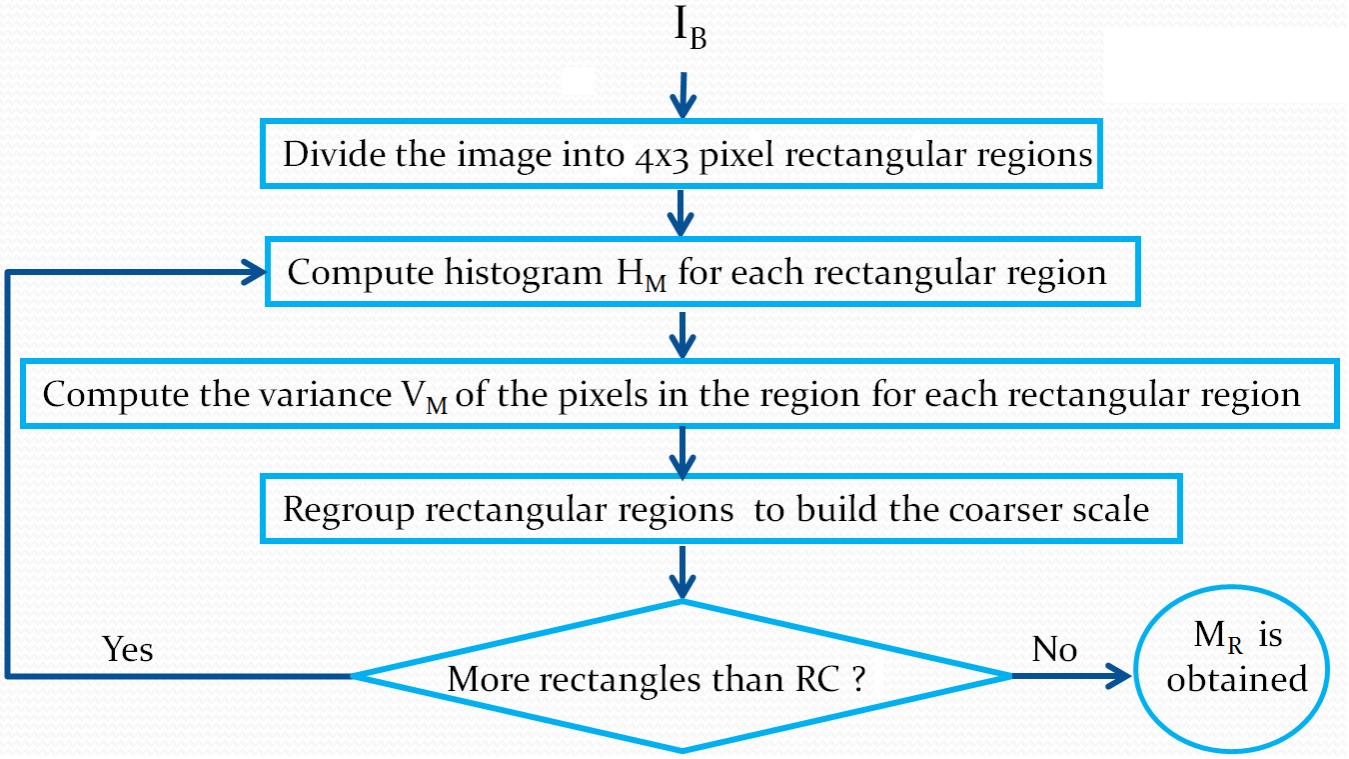
Steps of the background modeling process.

Our background model *M_R_* is simply based on statistics on the last RGB values of the pixels where no motion was detected. When we update the background, we substitute these pixels. We do this because color histograms account for pixels noise. Figure 2 illustrates the steps in the background modeling process.

The background model *M_R_* is built using rectangular region pixel statistics. According to Figure 2, the background image *I_B_* is first divided into 4 by 3 pixel rectangles. We selected this size because it corresponds to the aspect ratio of 4:3 images, which will be described at different scales using a rectangle hierarchy, where four rectangles of size 4 by 3 give rise at a coarser scale to a rectangle of size 8 by 6, and so on.

For each 4 by 3 rectangle (the finest scale), we compute two statistical measures. These measures will allow us to detect changes between *M_R_* and the current image at time *t* (*I_t_*). The first measure is the classical color histogram *H_M_*, and the second measure is the pixel intensity variance *V_M_* in the region, which is used to model region uniformity. This latter makes it possible to take into account illumination change (e.g., light shadows) that may affect histogram similarity. For example, two histograms might look different because of an intensity shift caused by a change of illumination. In that case, motion should not be detected. The pixel intensity variance allows us to modulate the histogram comparison threshold to make it more forgiving in the case of small illumination changes.

For color histograms, we use a uniform *K* bin quantification (usually *K* = 64) for each RGB channel. Variance is computed from the pixel intensities in the region. After these computations have been performed, we move to a coarser scale. We group together four 4 by 3 rectangles to construct 8 by 6 pixel rectangles. Each rectangle at the 4 by 3 level is used only once to create an overlapping 8 by 6 rectangle. The statistical measures at this new scale are computed by merging the statistics of the 4 by 3 rectangles. Rectangle grouping and statistic merging are performed until a user-defined number of coarse rectangles *R_c_* is obtained (usually *R_c_* = 100). Our background model *M_R_* is a set of statistical measures on rectangular regions at different scales.

#### 3.1.2. Motion detection

**Figure 3.**
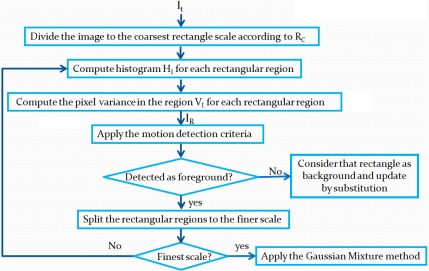
Steps in background updating and motion detection.

**Figure 4.**
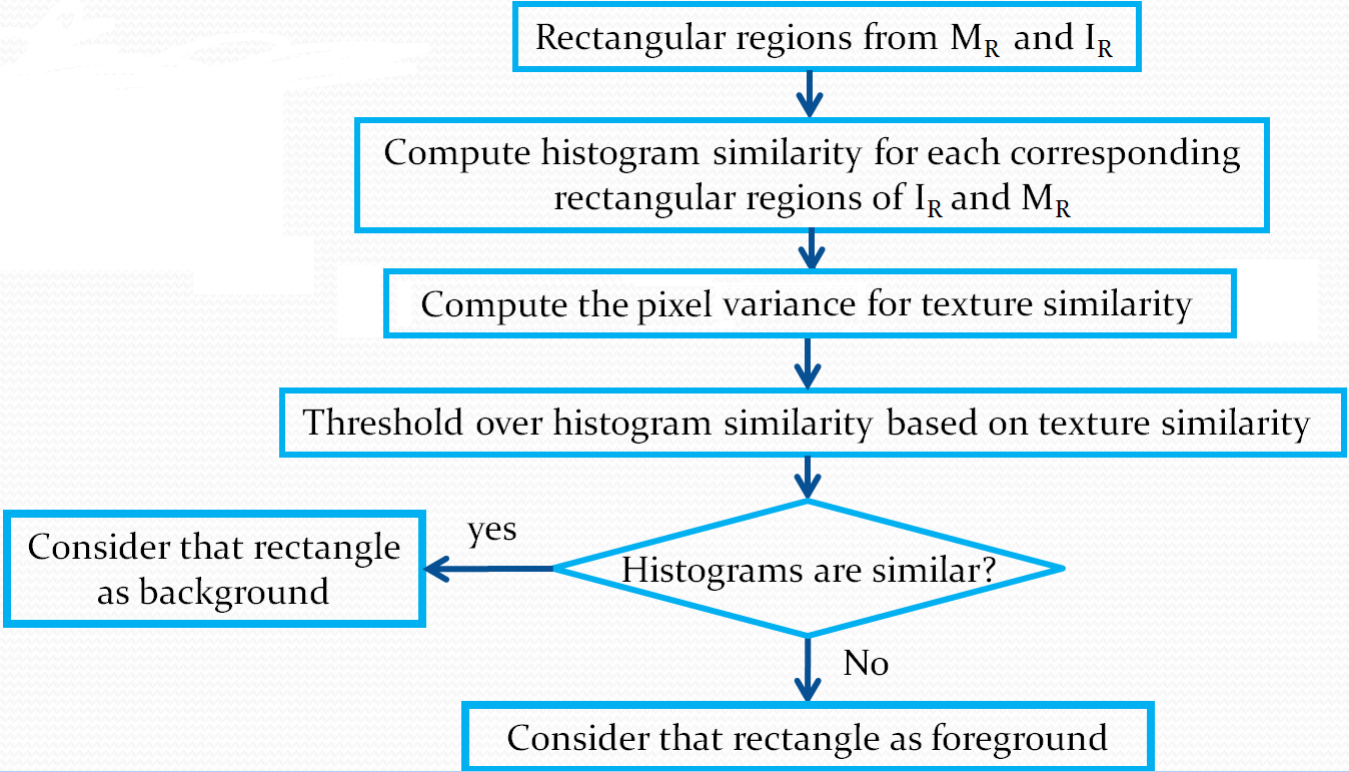
Motion detection criterion.

To detect motion, the current frame *I_t_* is modeled in the same way as the background by computing statistics on a rectangle set at different scales. The image model *I_R_* is obtained. The algorithm of this process is illustrated in Figure 3. The criterion to detect motion is shown in Figure 4. Differences between *I_R_* and *M_R_* are computed as follows.

**Figure 5.**
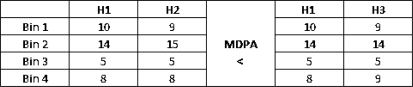
MDPA distance example. This distance is smaller for H1 and H2 than for H1 and H3. The Euclidean distances are similar.

**Figure 6.**
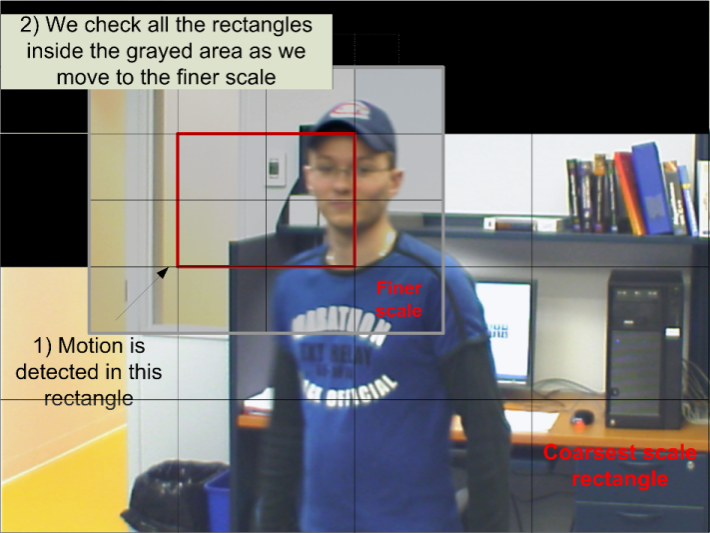
An example of motion detection as the algorithm progresses into scale. Black rectangles illustrate regions labeled as background. Motion is detected in the rectangle indicated. When, the algorithm moves to a finer scale, it will consider all the rectangles in the grayed area.

- Starting from the coarsest-scale rectangles and for each rectangle, we compute the histogram similarity using the MDPA distance [3] divided by the number of pixels in the rectangle. That is, the similarity *S_H_* for rectangle at position (*i, j*) is
  (1) $$S_{H}\;(H_{I}\;(i,j),H_{M}\;(i,j))=\frac{\sum_{b=0}^{K-1}\left|\sum_{k=0}^{b}\;(H_{I}\;(i,j)\;[k]-H_{M}\;(i,j)\;[k])\right|}{\sum_{b=0}^{K-1}H_{I}\;(i,j)\;[b]}$$
  where *H_I_*(*i, j*) and *H_M_* (*i, j*) denote histograms of the rectangles corresponding to *I_R_* and *M_R_*, and *b* and *k* are the *b^th^* and *k^th^* bin of the *K* bin histograms. MDPA distance is selected because similarly to the earth mover distance is a more accurate distance measure as it accounts for the error distribution among histogram bins. By error distribution, we mean that, given two bins that are different in two histograms, if these bins are close together in the histograms, then the contribution of this difference to the total distance will be smaller than if the bins are far apart. Thus, errors on nearby bins are considered smaller. Figure 5 illustrates that fact for histograms with similar Euclidean distances, but with different MDPA distances.
- Two histograms are similar if
  (2) $$S_{H}\;(H_{I}\;(i,j),\;H_{M}\;(i,j))<T$$
  where T is a similarity threshold. Figure 6 shows an example of this process over an image. As illustrated in Figure 6, if two histograms are similar, we simply copy the rectangle of *I_R_* into the background image to update it. In Figure 6, black rectangles correspond to rectangles copied to the background model, the moving object being the person. Otherwise, if the histograms are different, we move to the next finer scale to locate motion more precisely by considering smaller rectangles. To make sure of detecting moving regions located near a rectangle with detected motion, as we move to a finer scale, we also check neighboring rectangles (see Figure 6), as they may contain small regions connected to larger ones in other rectangles. These small regions might not cause a significant enough change in the previous-scale histogram. At all scales, the same computations are performed until the finest scale is reached. However, the threshold *T* is modified at each scale to take into account a smaller number of pixels. To avoid the influence of noisy pixels, as the rectangles get smaller, the threshold is increased by parameter Δ*T* (*i.e.*, *T* = *T* +Δ*T*).

#### 3.1.3. The role of the intensity variance

For greater robustness, a second statistical measure is used, which is the pixel intensity variance *V_M_* in the region. The purpose of this second measure is to model the uniformity in a rectangle and is similar to the measure used by Shen *et al.* [18]. The pixel intensity variance is also computed for each rectangle at all scales.

The intensity variance is used to modulate threshold *T* based on the probability of finding a moving object. Corresponding rectangle areas with different variances are cues that the probability of motion is higher.

Given a rectangle variance from the background model *V_M_* and the rectangle variance from the current image *V_I_*, a rectangle is said to be non-uniform if its variance is larger than threshold *T_v_*. If both rectangles are non-uniform, the following equation is applied to verify similarity:

(3) $$\mathrm{TS}=\frac{\left|\bar{{\mathrm{Vec}}_{M}^{T}}\cdot \bar{{\mathrm{Vec}}_{I}}\right|}{\left\|\bar{{\mathrm{Vec}}_{M}}\right\|\cdot \left\|\bar{{\mathrm{Vec}}_{I}}\right\|}$$

This gives a value between 0 and 1, corresponding to the rectangle texture similarity of the current rectangular region *Vec_I_* and the corresponding background region *Vec_M_* (both in vector format, that is, a region *N* × *M* is put into a vector 1 × *NM* row by row). $\bar{{\mathrm{Vec}}_{I}}$ is the 1 × *NM* vector |*μ_I_* − *Vec_I_*| and $\bar{{\mathrm{Vec}}_{M}}$ is the 1 × *NM* vector |*μ_M_* − *Vec_M_*|, where *μ_I_* and *μ_I_* are the mean pixel intensity of *Vec_I_* and *Vec_M_*, respectively. A result of 1 means that the two rectangles have exactly the same texture. Threshold *T* is adjusted by *T* = T* (0.8+*TS*). If the corresponding rectangles of *I_R_* and *M_R_* are both uniform, then most probably no changes took place, and we can be more stringent in histogram comparison. In this case, threshold *TS* = 1. If one rectangle of *I_R_* or *M_R_* is non-uniform, then it is assumed that a change occurred and *TS* = 0 (less stringent). The equation to adjust *T* was determined based on experiments.

### 3.2. Combination with Gaussian Mixture

Through testing, we have noted that our method is improved by combining it with the Gaussian Mixture [8] to obtain precise regions pixel level, as the regional precision obtained with our method is limited only by the smallest rectangle size, which is 4 by 3 pixels. Furthermore, Gaussian Mixture is improved as a preliminary segmentation has already been performed, which makes it more flexible (thresholds can be set higher to detect more pixels as foreground).

1. Gaussion Mixture method
  In the Gaussian Mixture method, RGB values measured in time at a given pixel position have been generated by a stationary process modeled by a Gaussian distribution
  (4) $$\eta (X_{t},\mu_{t},\sum_{t})=\frac{1}{{\left(2\pi \right)}^{n/2}{\left|\sum_{t}\right|}^{1/2}}e^{-\frac{1}{2}{\left(X_{t}-\mu_{t}\right)}^{T}\sum_{t}^{-1}\left(X_{t}-\mu_{t}\right)}$$
  where *X_t_* is a measure, *μ_t_* is the mean and ∑*_t_* is the covariance of the distribution [8]. Because the background may need to be represented by more than one distribution for each pixel, the background model is represented by *K* distributions. The probability that a measure *X_t_* belongs to the background is
  (5) $$P(X_{t})=\sum_{i=1}^{K}\omega_{i,t}*\eta (X_{t},\mu_{i,t},\sum_{i,t})$$
  where *ω_i,t_* is the *i^th^* distribution weight. In practice, it is assumed that each RGB channel is independent and of the same variance. Thus, a pixel position is modeled by *K* Gaussian distributions with a given mean *μ_t_* = [*μ_t,r_*, *μ_t,g_*, *μ_t,b_*] (one mean for each channel) and variance $\sigma_{t}^{2}$. A pixel *X_t_* is a moving pixel if
  (6) $$\left|X_{t}-\mu_{t}\right|>T_{g}\sigma_{t}$$
  for the *B* best distributions, where *T_g_* is a threshold to be fixed. Two additional parameters are the updating rate *α* for the distributions and *T_b_* for choosing the *B* best distributions among the *K* distributions.
2. Gaussion Mixture in our method
  In our method, Gaussian Mixture background subtraction is used in the following way. It is applied only when the finest scale is reached, which means that the Gaussian Mixture background model is updated for all pixels, but Gaussian Mixture motion detection is applied only for rectangles where motion is detected at the finest scale (4 × 3 pixels).

## 4. Experiments and Analysis

We have thoroughly characterized our method with various experiments. The goal of this work is to propose a background subtraction method that is efficient, but at the same time does not need too much parameter tuning to be easily applicable. Thus, the performance of RECTGAUSS-Tex was tested for parameter stability. The actual foreground/background segmentations were verified against ground-truth and with the same parameters for the videos in each dataset. In addition, RECTGAUSS-Tex was compared with other common background subtraction methods.

### 4.1. How We Tested

#### 4.1.1. Data and ground-truth

We used the Wallflower dataset[19], which includes ground-truth, to test our method in different situations (Bootstrap, Camouflage, Foreground aperture, Light switch, Moved object, and Time of day). This dataset is a couple of years old now, and the resolution is low 160 × 120). Thus, we shot new videos to test the algorithms with higher resolutions. The *IpCapAutoEx* video is a 320 × 240 sequence filmed at 7.5 fps, where an IP security camera (Sony SNC-RZ25N) is set in auto exposure mode. As a person walks by, the pixel colors change as the camera adapts to the scene content. The *BagPick-up* video is a 640 × 480 sequence filmed at 7.5 fps with a machine vision camera (Sony DFW-SX910), where a person picks-up a bag already present in the scene. Furthermore, the motion of the walker causes a curtain in the background to move. The *Walker* video is a 640 × 480 sequence filmed at 21.30 fps with an IP security camera (Sony SNC-RZ25N), where a walker enters a room, turns around, and then exits. The *AtriumLassonde* video is a 640x480 sequence filmed at 7.5 fps, where many walkers adopt random trajectories. The sequence is particularly noisy. We created about 6 ground-truth frames per video (total of 23) for these four videos.

#### 4.1.2. Description of the experiments

First, for the Wallflower dataset, seven different background subtraction methods were tested: RECTGAUSS-Tex (RGT), Single Gaussian (SG) [4] implemented by OpenCV, Gaussian Mixture (GM) [2], Temporal Average (TA) [5], Kernel Density Estimation (KDE) [14], CodeBook (CB) [15] and Texture-based Moving Object Detection (TBMOD) [1]. Second, for our own video files, we compared our method with GM, KDE, CB, and TBMOD. For each background subtraction method, there are some parameters to set. A suitable parameter set for a group of videos is the one that gives the largest number of correctly identified pixels for all videos. Accordingly, we calibrated all the methods except KDE and CB, to obtain that set. To perform the calibration, we swept the parameter space of each method. Since we were using two different datasets (Wallflower and our video files), we repeated this procedure to obtain two independent parameter sets (Table 1 and Table 2). TBMOD and KDE parameters are set to the recommended values by [1] and [20] (Table 3). For CB method, we used the default parameters of the implementation shown in Table 4 and found on the internet [21]. We were not able to sweep the parameter space and the papers of [14] and [15] do not propose any parameter set. We thus assumed that the default parameters are suitable ones. For TBMOD, we used the parameters recommended in [1].

For the performance evaluation of our proposed background subtraction method, we used two metrics: True Positive Rate (TPR) and False Positive Rate (FPR). True Positive (TP)/True Negative (TN) is defined as the number of foreground/background pixels that are correctly classified as foreground/background pixels. False Positive (FP)/False Negative (FN) is defined as the number of background/foreground pixels that are erroneously classified as foreground/background. The TPR and FPR are defined as

(7) $$\mathrm{TPR}=\frac{\mathrm{TP}}{\mathrm{TP}+\mathrm{FN}}$$

and

(8) $$\mathrm{FPR}=\frac{\mathrm{FP}}{\mathrm{FP}+\mathrm{TN}}$$

A high TPR will be obtained if the number of real foreground pixels detected in the extracted foreground is much larger than the number of real foreground pixels that are detected in the background (*TP* ≫ *FN*). Fewer parts of the real foreground are lost. The TPR only considers real foreground pixels, some of which will be detected as foreground and others as background. It is also important to investigate the influence of real background pixels in the extracted foreground. This can be computed with the false positive rate (FPR). If *TN* ≫ *FP*, the FPR value will be small, which means that most parts of the background are classified as background. In selecting the best method, it is important to look for a high TNR or a low FPR (*i.e.*, TNR = 1-FPR). The method with the highest TPR and the lowest FPR will be the best for background subtraction.

As shown in Table 1 and Table 2, there are often parameters to be set for background subtraction. The question is: is it possible to select a parameter set applicable for different videos? To find the answer, we performed an additional experiment to plot the normalized sensitivity curves for each parameter used in our method. These curves show the evolution of TPR and TNR against changes of each parameter in some predefined range. Because of normalization, we used the TPR and TNR values (*i.e.*, both should be high) instead of TPR and FPR. Our results and a discussion follow.

### 4.2. Results and Discussions

#### 4.2.1. Wallflower dataset

**Figure 7.**
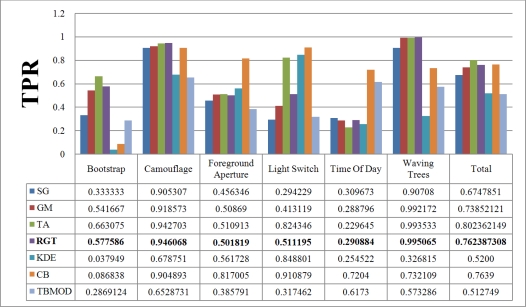
True Positive Rate of various background subtraction methods for the Wallflower dataset. The Total column represents the combination of all videos. SG: Single Gaussian, GM: Gaussian Mixture, TA: Temporal Average, RGT: our method, KDE: Kernel Density Estimation, CB: CodeBook, TBMOD: Texture-based Moving Object Detection.

**Figure 8.**
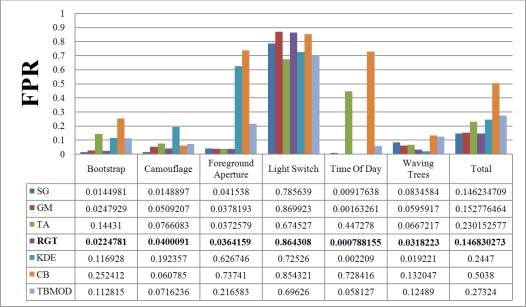
False Positive Rate of various background subtraction methods for the Wallflower dataset. The Total column is the combination of all videos. SG: Single Gaussian, GM: Gaussian Mixture, TA: Temporal Average, RGT: our method, KDE: Kernel Density Estimation, CB: CodeBook, TBMOD: Texture-based Moving Object Detection.

Figure 7 and Figure 8 show the TPR and FPR obtained for the Wallflower dataset with the seven background subtraction methods. The parameters used for each method are listed in Table 1. Recall that the best method should have the highest TPR value. The Single Gaussian method, TBMOD, and the Kernel Density Estimation methods have the lowest TPRs in general for all the videos (according to their Total columns). Temporal Average, CodeBook, and RECTGAUSS-Tex have the highest TPR respectively. The TPR of Gaussian Mixture method, which is commonly used in computer vision tasks, are often smaller than these latter three methods.

For complete separation of foreground from background, the FPR value has to be very small (*i.e.*, ideally equal to zero). Indeed, if this value is not small, it means that most parts of the image have been detected as foreground, and the background subtraction method is not appropriate because it does not label background pixels correctly. Figure 8 shows the FPR results. CodeBook has the highest FPR, and that of our method is among the smallest in all the videos and all the methods considered. This shows that our method combines a high TPR and a small FPR. The behavior of the Gaussian Mixture method is similar, however it is outperformed by our method. Because of intensity changes affecting individual pixels (texture is computed based on a central pixel), the TBMOD method does not perform well in general, as region properties are based on a single pixel.

**Figure 9.**
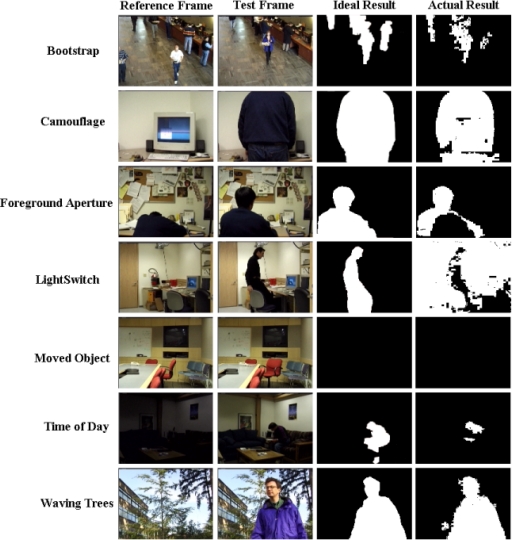
Detection results of the proposed method for the Wallflower dataset.

There is no motion in the *Moved Object* video of the Wallflower dataset. Thus, the TPR and FPR values of all background subtraction methods are equal to zero. This is why this result is not shown in the figures. Based on the TPR and FPR values, all the methods are very sensitive to a sudden change of illumination. For this event, the Temporal Average method has the highest TPR and the smallest FPR. Figure 9 shows the results of the proposed background subtraction method for the Wallflower dataset ground-truth frames.

In the second experiment, we tested our method with our dataset. The parameters of each method are listed in Table 2. Rectangle size depends on the size of the objects to detect. Fewer rectangles mean larger ones at the coarsest level, and thus more filtering of small motions. In our videos and for the Wallflower dataset, the moving objects are relatively large. This is why 100 rectangles are enough at the coarsest level.

#### 4.2.2. Our dataset

**Figure 10.**
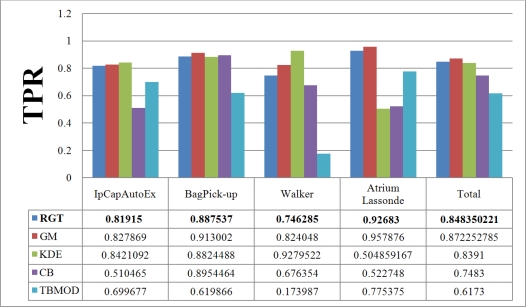
True Positive Rate of different variations of the proposed background subtraction method for our dataset. The Total column is the combination of all the videos. RGT: our method, GM: Gaussian Mixture, KDE: Kernel Density Estimation, CB: CodeBook, TBMOD: Texture-based Moving Object Detection.

**Figure 11.**
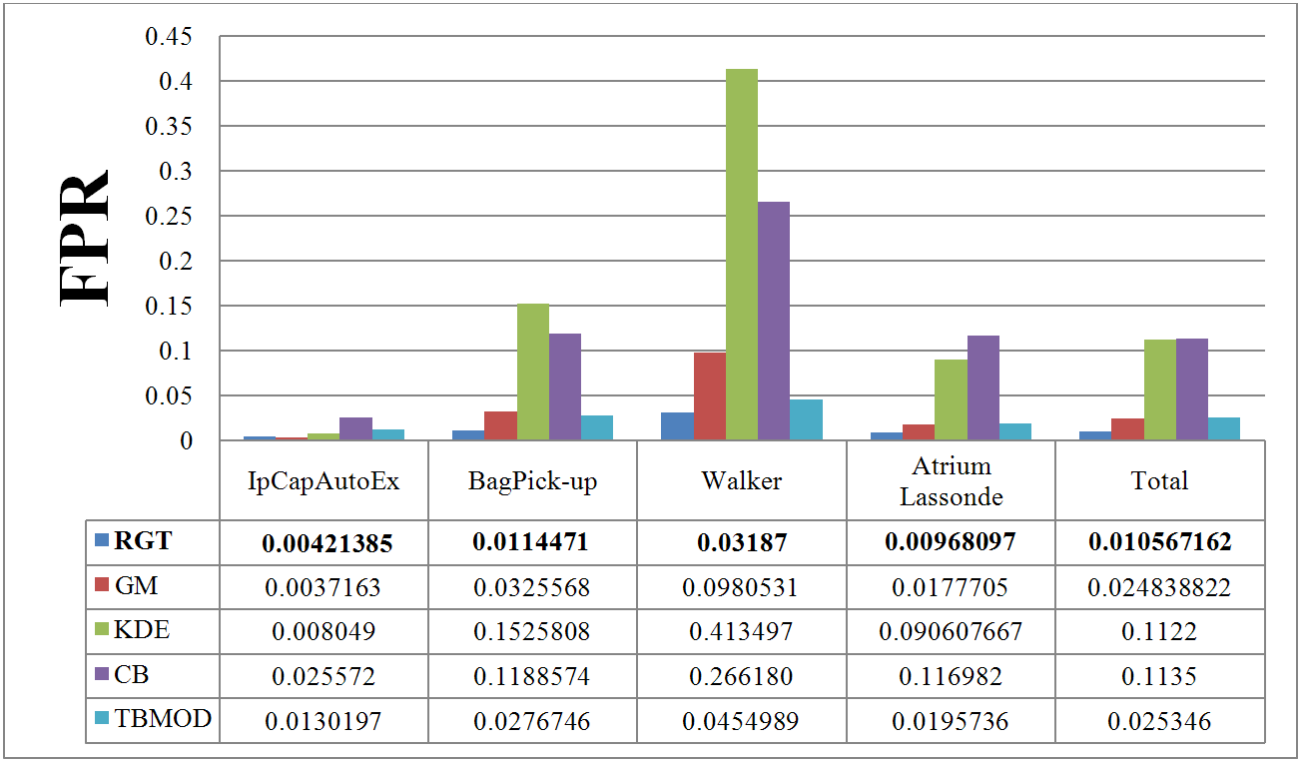
False Positive Rate of variations of the proposed background subtraction method for our dataset. The Total column is the combination of all videos. RGT: our method, GM: Gaussian Mixture, KDE: Kernel Density Estimation, CB: CodeBook, TBMOD: Texture-based Moving Object Detection.

**Figure 12.**
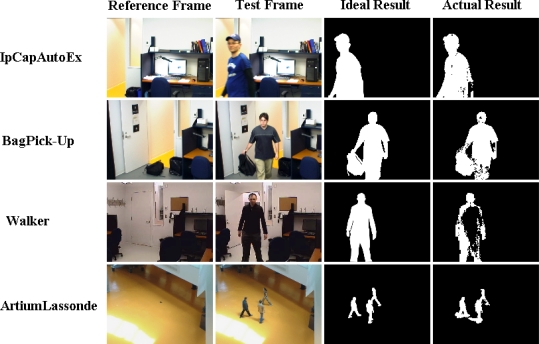
Detection results of the proposed method for our dataset.

**Figure 13.**
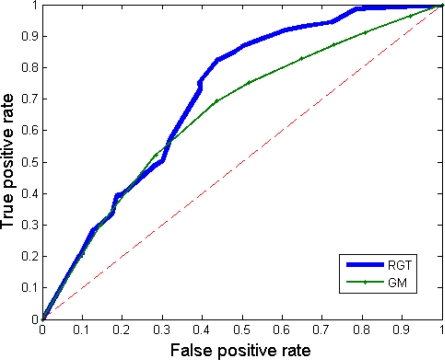
ROC curve of our method (parameter *T*) and Gaussian mixture (parameter *Tg*) for all the tested videos.

Figure 10 and 11 give the TPR and FPR for each video of our database and Figure 12 shows the detection results of the proposed method for our dataset. There is also a light switch event for the *Walker* video, causes higher FPRs in the results. In addition, we computed the total TPR and FPR for these four videos. A similar situation is to have a video that is a combination of various video categories. Here again, the Gaussian Mixture method, our method, and the Kernel Density Estimation method have the highest TPR. However, as discussed previously, the FPR of all of them, except our method, TBMOD, and GM, are high. The RECTGAUSS-Tex method has the smallest FPR. Thus, using the combination of both the texture property and the Gaussian function in our method makes RECTGAUSS-tex an improvement over the Gaussian Mixture method, because it has a roughly similar TPR, but a smaller FPR. This means that fewer errors are included in the foreground. This is confirmed by plotting the receiver operating characteristic (ROC) curve of both methods Figure 13. To plot the ROC curve, parameter *T* of RGT and parameter *Tg* of Gaussian mixture have been varied, and all videos (our dataset and wallflower dataset) have been processed to obtain the TPR and FPR. The ROC curve confirms that RGT classify better pixels of an image when a TPR of 0.6 or more is desired.

#### 4.2.3. Parameter stability

Next, we tested the effect of parameters on the performance of the proposed background subtraction method. This can be determined by a metric called sensitivity [1]. The TPR and FPR curves are obtained from some predefined ranges of parameter variations. Because of the closeness of the TPR, they are normalized according to this formula:

(9) $$\mathrm{NTPR}=\frac{\mathrm{TPR}-\min(\mathrm{TPR})}{\max(\mathrm{TPR}-\min(\mathrm{TPR}))}$$

**Figure 14.**
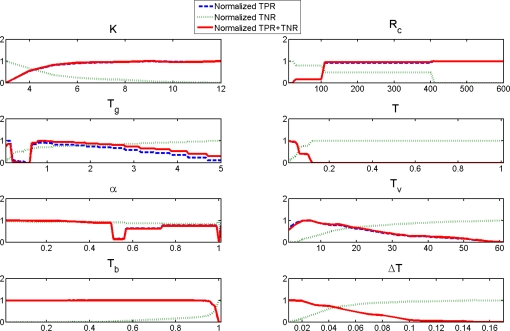
Normalized TPR, TNR and TPR + TNR of each parameter of the proposed background subtraction method for the *ArtiumLassonde* video. Untested parameters were kept fixed.

The same normalization is performed for the TNR. As shown in Figure 14, the crossing point of the curves is the optimum point with an equal tradeoff between TPR and FPR.

Now, are the results stable for some parameter range? Do we have to change all the parameters or just a few? Do we have the parameter insensitivity that we wished for? To answer these questions, the normalized sum of TPR and TNR is also calculated to arrive at a general metric for evaluation. As shown in Figure 14, many parameters give a stable TPR+TNR over a wide parameter value range. Exceptions are the *T* (histograms similarity threshold) and Δ*T* (rectangle similarity threshold increase) parameters that need more tuning. Thus, we have only partially achieved our goal of having a method that does not require parameter tuning. Note that for *R_c_*, because of the 4:3 ratio of the images, the possible values for this parameter are 25, 100, 400, 800, *etc*.

The selection of the best parameter set also depends on the application. For some applications, having a high TPR is much more important than a high TNR, because there is a requirement not to miss any foreground parts (e.g., hand tracking, face tracking, *etc*.). With a high a TPR, most parts of the real foreground are classified correctly. In some other cases, having high TNR is more important, because perturbations like shadows, waving trees, periodic motion, *etc.*, need to be eliminated.

Table 5 shows the frame processing rate (in frames per second) of the Gaussian Mixture method and ours, for different resolutions. These algorithms were implemented on an Intel Xeon(R) 5150 in C++ using OpenCV. Compared to the Gaussian Mixture method, our algorithm is slower by 3% for the 640 × 480 videos, slower by 4% for the 320 × 240 videos and slower by 6% for the 160 × 120 videos. Thus, the overhead required to process videos by first using our region-based method is small. For the proposed algorithm, we obtained 7.1 fps for the processing of 807 frames with 640 × 480 image resolutions (our dataset), 27.2 fps for 1,500 frames with 320 × 240 image resolutions and 81.5 fps for 16,158 frames with 160 × 120 image resolutions. Our method can be used for online applications with image resolutions of 320 × 240 or less with our unoptimized implementation (the same as for the Gaussian Mixture method). The performance hit is mostly caused by the MDPA distance which is much slower than the Euclidian distance for comparing histograms. We selected MDPA because it is a more accurate distance measure as it accounts for the error distribution among histogram bins. By using Euclidian distance, we get a faster processing rate, but the results are not as good as with MDPA.

### 4.3. Results Summary

The results show that our method has the following advantages over to the state-of-the-art:

- Because of the use of rectangular regions, local noise affecting individual pixels is filtered naturally by the histogram properties. Waving leaves in a background can thus be dealt with directly during foreground detection;
- Small objects can be ignored by customizing the size of the rectangles at the coarsest level (e.g., to detect cars, not humans);
- Texture and rectangular region intensity variance allow our method to deal with light shadows and small illumination changes by adjusting the foreground detection threshold when comparing histograms;
- Motion detection can be performed by selecting a different scale dynamically at each frame by stopping histogram subdivision at the foreground detection step. Foreground object shapes may be detected with precision only when required during a tracking process (see Figure 1).

However, like many other background subtraction methods, our method does not handle large illumination changes. Perhaps, this could be accounted for by considering histogram shifts. Another drawback is the choice of the coarsest rectangle size, which needs to be selected to be small enough to detect the object of interest. Objects to detect must occupy a large enough proportion of the coarsest rectangle. Thus, balancing the rectangle size for the detection of small objects while filtering noise might be incompatible for very small objects.

## 5. Conclusions

In this paper, a novel background subtraction method is proposed. The background is modeled by rectangular regions described by a color histogram and a texture measure. It is modeled at different scales to detect motion more and more precisely. This background model is combined with the Gaussian Mixture model. The use of rectangular regions filters out small motions like swaying vegetation, and data acquisition noise. The Gaussian Mixture background subtraction method then completes the work by detailing the foreground detection in rectangular areas where significant motion is detected. Compared to the Gaussian Mixture method alone, RECTGAUSS-Tex gives less false positive detection for similar true positive results.

The algorithm was evaluated with various videos against different illumination changes and resolutions. The results obtained show that RECTGAUSS-Tex outperforms the Gaussian Mixture method as it has a similar TPR, but a smaller FPR. For the datasets used, it also outperforms CodeBook, KDE, and TBMOD using their default parameters. Although our algorithm uses eight parameters, six of them are stable, and only two requires tuning.

Our motion detection algorithm can be performed at different scales to adjust to the object shape precision needed for one application, which means that we can perform detection only at coarse scale with large rectangles and without using the Gaussian Mixture method.

The drawback of the method is that it requires 3% to 6% more processing than Gaussian Mixture method, but, like the Gaussian Mixture method, ours is appropriate for online application with an image resolution of 320 × 240 or less with our implementation. However, MDPA distance calculations could be performed in parallel to speed up processing. Future work will involve adjusting the thresholds dynamically based on scene content and object appearance. So, if the color of an object is similar to the background, the detection threshold could be decreased in this background area for more sensitivity.

## Figures and Tables

**Table 1.** Parameters for the experiment using Wallflower dataset.

Method	*K*	*T_g_*	*α*	*T_b_*	*T*	Δ*T*	*R_c_*	*T_v_*	*E*
SG	-	-	0.05	-	-	-	-	-	-
GM	3	3.5	0.007	0.50	-	-	-	-	-
TA	-	-	-	-	-	-	-	-	31

RGT	5	3.0	0.005	0.30	0.044	0.014	100	2	-

*K*: Number of Gaussian distributions, *T_g_*: Foreground/background threshold (in number of standard deviation), *α*: Learning rate, *T_b_*: Background distribution proportion, *T*: Rectangle similarity threshold, Δ*T*:Rectangle similarity threshold increase, *R_c_*: Number of rectangles, *T_v_*: Rectangle non-uniformity similarity threshold, *E*: Similarity threshold (in RGB level).

**Table 2.** Parameters for the experiment using our dataset.

Method	*K*	*T_g_*	*α*	*T_b_*	*T*	Δ*T*	*R_c_*	*T_v_*
GM	3	3.4	0.009	0.50	-	-	-	-
RGT	5	3.0	0.01	0.30	0.38	0.0095	100	2

*K*: Number of Gaussian distributions, *T_g_*: foreground/background threshold (in number of standard deviation), *α*: Learning rate, *T_b_*: Background distribution proportion, *T*: Rectangle similarity threshold, Δ*T*:Rectangle similarity threshold increase, *R_c_*: Number of rectangles, *T_v_*: Rectangle non-uniformity similarity threshold.

**Table 3.** Parameters for the experiment using both datasets.

Method	*K*	*T_B_*	*α_b_*	*α_ω_*	*T_P_*	*R_Region_*	*R*	*P*	*OS*	*m*	*PS*
TBMOD [1]	3	0.4	0.01	0.01	0.65	9	2	6	-	-	-
KDE [20]	-	-	-	-	-	-	-	-	10	15	1e-20

*K*: Number of histograms, *T_B_*: Threshold over sum of weights for B first histogram, *α_b_*: Learning rate, *α_ω_*: Learning rate, *T_P_*: Threshold for proximity measure, *R_region_*: Region radius, *R*: Neighborhood radius, *P* : Neighborhood size, *OS*: Object sensitivity, *m*: Object size, *PS*:Probability sensitivity.

**Table 4.** Parameters for the experiment using both datasets.

Method	*ε*_1_	*ε*_2_	*α*	*β*	*Period*(%)	*n_r_*	*C_t_*	*b_s_*
CB [21]	20	20	0.7	1.2	60	3	20	100

*ε*_1_: Color sampling bandwidth, *ε*_2_: Color detecting threshold, *α*: Shadow bound, *β*: Highlight bound, *Period*: Max negative run-length period, *n_r_*: Spot noise removal over a 3 × 3 box, *C_t_*: Color tolerance, *b_s_*: Blob size.

**Table 5.** Processing rate (fps) of various background subtraction methods for different image resolutions.

Resolution	Processing rate of GM (fps)	Processing rate of RGT (fps)
640 × 480	7.33	7.1
320 × 240	28.3	27.2
160 × 120	86.4	81.5
